# Active Components of *Leptospira* Outer Membrane Protein LipL32 to Toll-Like Receptor 2

**DOI:** 10.1038/s41598-017-08743-y

**Published:** 2017-08-21

**Authors:** Shen-Hsing Hsu, Cheng-Chieh Hung, Ming-Yang Chang, Yi-Ching Ko, Huang-Yu Yang, Hsiang-Hao Hsu, Ya-Chung Tian, Li-Fang Chou, Rong-Long Pan, Fan-Gang Tseng, Chih-Wei Yang

**Affiliations:** 1 Department of Nephrology, Kidney Research Center, Chang Gung Memorial Hospital, Chang Gung University College of Medicine, Taoyuan, 33333 Taiwan, ROC; 2 Department of Life Science and Institute of Bioinformatics and Structural Biology, College of Life Science, National Tsing Hua University, Hsin Chu, 30013 Taiwan, ROC; 3 Department of Engineering and System Science, College of Nuclear Science, National Tsing Hua University, Hsin Chu, 30013 Taiwan, ROC

## Abstract

Proteins belonging to the toll-like receptor (TLR) family, particularly TLR2, are the major components of innate immunity against *Leptospira* infection. The ligands for TLR2 harbor several conserved patterns such as lipidation molecules, leucine-rich repeat (LRR) domains, TLR2 binding motifs, and TLR2 binding structure. In *Leptospira*, LipL32 interacts with TLR2 on human kidney cells concomitantly stimulating inflammatory responses. However, the binding mechanism of LipL32 to TLR2 is unknown. The computational prediction suggests that β1β2, loop-α3-loop, and α4 domains of LipL32 play vital roles in LipL32-TLR2 complex formation. To test these predictions, protein truncation experiments revealed that LipL32ΔNβ1β2 significantly decreased the affinity to TLR2 while LipL32ΔCα4 slightly reduced it. Interestingly, LipL32ΔCenα3 retained affinity to TLR2 in the absence of Ca^2+^ ions, indicating that Cenα3 play a role preventing the interaction between LipL32 and TLR2. Furthermore, the critical residues of LipL32 involved in interacting with TLR2 suggested that V35S, L36S and L263S variants significantly decreased the affinity to TLR2. The results further confirm that LipL32 interacts with TLR2 through Nβ1β2 and Cα4 domains of LipL32 as well as LipL32-TLR2 complex formation results from hydrophobic interactions. This study provides a detailed mechanism of the interaction between LipL32 and TLR2 and the residues involved in complex formation.

## Introduction

Leptospirosis is an overlooked risk in acute and chronic kidney diseases, which contributes to high mortality and morbidity worldwide, particularly in warm and humid areas^1–5^. Humans become infected through contact with *Leptospira* containing water, food, soil or urine from infected animals through exposed mucosal surfaces or broken skin^1, 6^. Clinical manifestations of Leptospirosis range from mild to severe or even potentially lethal as characterized by high fever, intense jaundice, bleeding, renal and pulmonary dysfunction, neurologic alterations, and cardiovascular collapse^7^. Kidneys are one of the main target organs of *Leptospira*, especially the proximal tubule epithelial cells (PTECs), resulting in tubulointerstitial nephritis and acute renal failure^8^.

Pathogens have developed a variety of mechanisms to survive in the host environment and pathogenic species of *Leptospira* have evolved to complete the infectious cycle, which includes entering the host, evading the immune responses, adhering and colonizing to tissues, and finally exiting the host to initiate a new infection, while maintaining the capacity to survive in the environment^9^. At the adhesion stage, outer membrane components mediate the major interactions between the pathogen and the host cell. The important outer membrane components from *Leptospira* potentially involved in virulence have been recognized including major outer membrane lipoprotein 32 (LipL32)^10^, lipopolysaccharides (LPS)^11^, *Leptospira* immunoglobulin-like proteins (Lig)^12^, *Leptospira* endostatin-like proteins (Len)^13, 14^, and *Leptospira* OmpA-like lipoprotein (Loa22)^15^. Notably, the most abundant outer membrane component found in pathogenic *Leptospira* is LipL32, a lipoprotein with a lipid modification at its Cys^20^ residue and a signal peptide tag at its N-terminus^16–18^. The crystal structure of LipL32 reveals a jellyroll-fold structure and a calcium ion was shown to be important for structural and thermal stability^19–21^. In addition, LipL32 has been validated by the affinity to different components of extracellular matrix (ECM), including collagen I, collagen IV, collagen V, laminin, and plasma fibronectin while the C terminal and intermediate domain of LipL32 are responsible for the interaction to ECMs^9, 22^. In addition, purified LipL32 protein increased the permeability and decrease the expression of zonula occludens-1 (ZO-1) and F-actin in human umbilical vein endothelial cells (HUVEC)^23^. Recently, post-translational modification (PTM) of LipL32 was observed to include either acetylation or tri-methylation of lysine residues within multiple peptides, and PTM was further demonstrated to be related to immune-reactivity^24^. These evidences demonstrate that LipL32 stimulates inflammatory responses, however, the exact mechanism is still unclear. Our previous studies demonstrated that LipL32 induces inflammatory signals through the innate immune component, Toll-like receptor 2 (TLR2)^8, 25, 26^. Among these inflammatory signals, chemotactic chemokines play vital roles in the recruitment of macrophage or neutrophil to the site of inflammation and the level of these chemotactic factors are up-regulated in a variety of glomerular and interstitial renal injuries^27^. IL-8 is a chemokine contains ELR (glutamic acid-leucine-arginine) motif with ability to induce neutrophil activation and MCP-1 is monocyte chemoattractant protein-1 that is secreted by several cell types including renal cells^27, 28^. Tumor necrosis factors alpha (TNF-α) and matrix metalloproteinase-7 (MMP-7) have been demonstrated to up-regulate when treatment of LipL32 to renal cells^25^. In additions, other macrophage chemotatic factors including CXCL1/GRO-α and Osteopontin (SPP1) are also selected to measure the renal interstitial inflammation^27, 29, 30^.

TLR family proteins play a pivotal role in innate immunity by recognizing conserved patterns in diverse microbial molecules^31^. Association of TLR2 with TLR1 or TLR6 is essential for sensing bacterial lipoproteins and lipopeptides^32, 33^. TLRs containing leucine-rich repeats (LRRs) are responsible for pattern recognition of the extracellular portion and a Toll/IL-1 receptor (TIR) domain is responsible for signal transduction through the cytoplasm^34^. The crystal structure of TLR2/1 complex with its ligand, Pam_3_CSK_4_, revealed that two fatty esters of the glycerol moiety are embedded into a hydrophobic pocket of TLR2, while the amide-bound lipid chain fits into the hydrophobic channel of TLR1^35^. The lipid molecule of lipoprotein presumably supports the binding domain of TLR2. Recently, the complex structure of TLR2 in association with staphylococcal superantigen-like protein 3 (SSL3) revealed that the interaction is predominantly mediated by hydrophobic contacts between the two proteins^36^. In addition, the PorB protein from *Neisseria meningitidis* was suggested to act as a TLR2 ligand with the binding mechanism hypothesized to involved electrostatic interactions that contribute to ligand/receptor interaction^37^. Moreover, the BspA protein from *Tannerella forsythia* which contains a LRR domain, was also suggested to be a TLR2 ligand^38^. Furthermore, the pentameric B subunit of type IIb *E. coli* enterotoxin (LT-IIb-B_5_) uses its hydrophobic upper-pore region to directly bind TLR2 and the ring with four residues (Met^69^, Ala^70^, Leu^73^, and Ser^74^) are considered to be TLR2-binding sites for induction of inflammatory responses^39^. These TLR2 ligands use various binding mechanisms to interact with innate immune components that induce inflammatory responses. In *Leptospira*, it has been proven that the LipL32 stimulated inflammatory responses through TLR2 and the calcium binding cluster (including Asp^132^, Thr^133^, Asp^164^, Asp^165^, and Tyr^178^) of LipL32 can regulate the affinity between LipL32 and TLR2 during infection with *Leptospira* ^4, 25, 26^. However, the TLR2 binding domains and mechanisms for binding with LipL32 are unclear. Accordingly, identification of the binding mechanisms for LipL32-TLR2 interaction will provide new insights into innate immunity.

In this study, we predicted the LipL32-TLR2 complex from the Cluspro website and the binding domains were confirmed by truncation experiments. We demonstrated that the active components of LipL32, including N terminal β1β2 (Nβ1β2), central loop-α3-loop (Cenα3), and C terminal α4 helix (Cα4) domains, play critical roles involving in TLR2 interaction. In combination with site-directed mutagenesis and protein-protein interaction studies, we find the essential residues within active components of LipL32 participating in TLR2 binding. Our study suggested a novel working mechanism of a potential TLR2 ligand.

## Results

### Molecular docking

**Figure 1:**
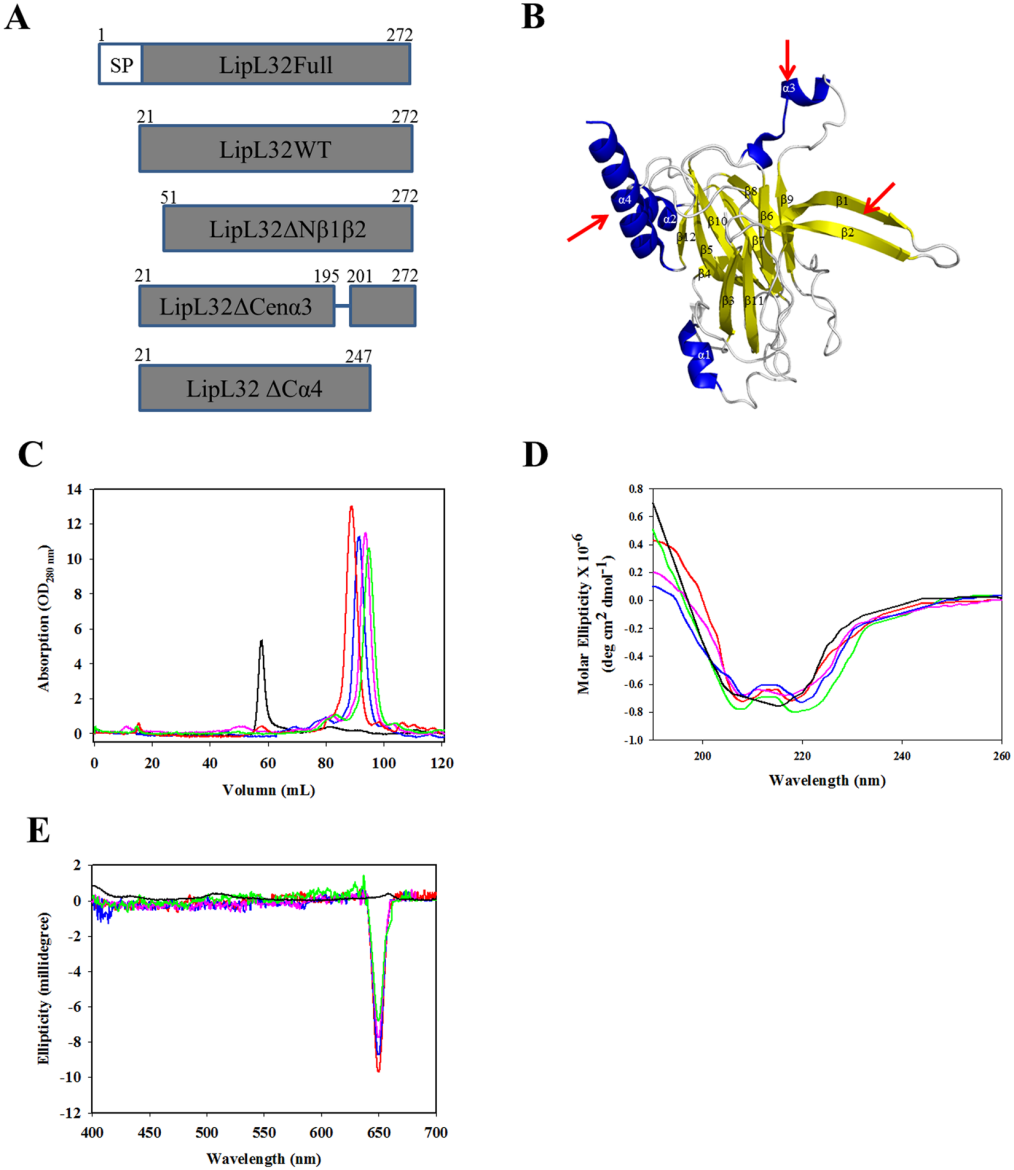
Characterization of LipL32 truncation variants. (**A**) The cartoons show the construction of LipL32 truncation variants. SP, signal peptide. (**B**) Three dimension structure of LipL32 and its relative domains. Blue, α-helix; Yellow, β-sheet; Gray, random-coil; red arrows, active motifs for TLR2 interaction. (**C**) Size exclusion chromatography (SEC) of purified TLR2 protein and LipL32 variants. TLR2 protein and LipL32 variants were purified as described in Materials and Methods and SEC was used to identify the integrity and purity of these proteins. (**D**) Circular dichroism (CD) of purified TLR2 protein and LipL32 variants purified. The secondary structure of purified TLR2 protein and LipL32 variants were measured by CD spectroscopy and the spectra were record between 260 nm and 190 nm. (**E**) Ca^2+^ binding assay by stains-all assay. The Ca^2+^ binding activity of LipL32 variants were tested by stains-all and the J band at 660 nm was measured. TLR2 protein was used as negative control. Black, TLR2 protein; Red, LipL32WT; Blue, LipL32ΔCenα3; Pink, LipL32ΔCα4; Green, LipL32ΔNβ1β2 in Figure (**C**–**E**).

The crystallographic structure of the TLR2/1-Pam_3_CSK_4_ complex suggests a mechanism for recognition of lapidated ligands by TLRs^35^; however, the protein ligands of TLR2 are still unknown. To identify the binding mechanism, we modeled the interaction between LipL32 (2WFK) and TLR2 (2Z7X). The top ten scoring models of TLR2/LipL32 were retrieved from ClusPro^40^ protein-protein docking website and were divided into three groups on the basis of LipL32 orientation in interact with TLR2 (Fig. S1A). According to the three groups, LipL32 may interact with TLR2 through its central domain (Model I), N terminal domain alone (Model II), or N terminal domain assisted by the C terminal domain (Model III). From the results of protein docking predictions, three vital domains (including the N terminal β1β2 sheet, central α3 helix, and C terminal α4 helix) were predicted to be involved in interaction with TLR2. Therefore, these three domains were individually deleted in construction of the truncation variants for protein expression and to assay their affinity to TLR2 (Fig. 1A). The three vital domains were shown in the 3D structure as indicated in Fig. 1B (red arrows).

### Protein construction, purification, and characterization

TLR2 protein is type I transmembrane protein with three domains including leucine-rich repeat (LRR), transmembrane domain (TMD), and Toll/interleukin-1 receptor domain (TIR)^31, 34^. The LRR domain is responsible for sensing the bacteria components and consequently dimerizes with other LRR domain of TLR1, 2, or 6 to induce downstream signaling. Therefore, the human full-length *tlr2* DNA was cloned into the vector pLenti6.3/V5-TOPO with the primers listed in Table 1. The DNA sequence was further confirmed by DNA sequencing. The pLenti6.3/V5-TLR2 DNA was packaged using the ViraPower™ Lentiviral Expression System according to the manual suggestion and transfected into HEK293FT cell for stable cell line construction (Invitrogen). After selecting by blasticidin, HEK293-TLR2 cell were cultured in the medium of DMEM with 10% FBS and maintained at 37 °C in a humidified atmosphere containing 5% CO_2_. In the protein purification process, the HEK293-TLR2 cell were solublized in the PBS buffer containing 0.5% CHAPS with gently stirred for 1h at 4 °C. Recombinant TLR2 (rTLR2) protein was purified in a series of purification procedures including anti-V5 antibody activated NHS resin affinity purification, mono Q ion exchange column, and Superdex 200 gel filtration column (Fig. S2A). The molecular mass of purified rTLR2 protein was about 240 kDa as determined by using Superdex 200 gel filtration column (Fig. 1C). The function of rTLR2 protein was tested by the affinity to its nature ligands including lipoteichoic acid (LTA), Pam_3_CSK_4_, and peptidoglycan (PGN), respectively^25^ (Fig. S3). The interaction of the purified rTLR2 protein with its nature ligands showed statistically significant difference indicated that the purified rTLR2 protein is suitable for the ligands selection.

**Table 1:** Primers used in this study.

Genes	Forward (5→3′)	Reverse (5′→3′)
TNF-α	ATGAGCACAGAAAGCATGATCCGC	CCAAAGTAGACCTGCCCGGACTC
CXCL8/IL-8	ATGACTTCCAAGCTGGCCGTGGCT	TCTCAGCCCTCTTCAAAAACTTCTC
CCL2/MCP-1	CCGCTGTTATAACTTCACC	ACATCCCAGGGGTAGAACTG
hMMP7	TGAGCTACAGTGGGAACAGG	CATCGAAGTGAGCATCTCC
hTLR2	CGACGCGTAGCATGCCACATAC	GCACGCGTGGACTTTATCGCA
LipL32WT	TTACCGCTCGAGGTGCTTTCGGTGGTCTGC	TGTTAACCCGGGTTACTTAGTCGCGTCAGA
LipL32ΔNβ1β2	TTACCGCTCGAGGTCTTCCCTACGGATCTG	TGTTAACCCGGGTTACTTAGTCGCGTCAGA
LipL32ΔCenα3	AACTCTCTTACTAGAATCAAG	ATAAGTATCGTCACCATC
LipL32ΔCα4	TTACCGCTCGAGGTGCTTTCGGTGGTCTGC	TGTTAACCCGGGTTATGCAACGAAAGATCC
F34S	AAAAAGCTCTAGTGTTCTGAGCGAGGACACAATC	AGGCTTGGCAGACCACCG
V35S	AAGCTCTTTTAGTCTGAGCGAGGACAC	TTTAGGCTTGGCAGACCAC
L36S	CTCTTTTGTTAGTAGCGAGGACACAATC	CTTTTTAGGCTTGGCAGAC
D195A	AAATCTTTTGCCGATCTGAAAAACATC	TGGAGGATTAGGGATCTTG
D196A	TCTTTTGACGCTCTGAAAAACATC	TTTTGGAGGATTAGGGATC
L263S	TGAAGAGTCTAGCAAAAAAGCTGCTTC	GCAGCGATAGCTTGTTTTTG

The full-length LipL32 protein containing 272aa with N terminal 20 signal peptide. The mature form of recombinant LipL32 protein (rLipL32; 21–272) and the different fragments including rLipL32ΔNβ1β2 (51–272), rLipL32ΔCenα3 (deletion the residues of 195–200), and rLipL32ΔCα4 (21–247), respectively, were constructed according to the modeling results and these variants were used to evaluate the affinity to TLR2 (Fig. 1A). In addition, the vital residues within the deletion domains of rLipL32 that potentially involved in TLR2 interaction including F34S, V35S, L36S, D195A, D196A, and L263S were performed by using Q5® Site-Directed Mutagenesis and the primers used for site-directed mutagenesis were listed in Table 1. The rLipL32WT and its variants were expressed in the *E*. *coli* ClearColi^TM^ BL21(DE3) and BL21(DE3)pLys to obtained the recombinant proteins (Fig. S2B and C). The recombinant proteins were purified by Ni^2+^-NTA affinity column and eluted in 250 mM imidazole. In order to determine the endotoxin contents, *Limulus* amebocyte lysate (LAL) assay was used to measure the endotoxin contamination of these recombinant proteins. The rLipL32 proteins from *E. coli* BL21(DE3)pLys contained high level of endotoxin that >100000EU/mg, while the protein from *E. coli* ClearColi^TM^ BL21(DE3) significant decreased the endotoxin contamination to 688.5 ± 23.4 EU/mg (Fig. S4). The recombinant proteins from ClearColi^TM^ BL21(DE3) contain relatively low endotoxin and suitable for mammalian cell applications. In order to improve the purify and removed endotoxin contamination of the recombinant proteins, Mono Q ion exchange chromatograph was further used to purify the LipL32 protein and its variants as well as the proteins were assayed by Superdex 200 gel filtration column (Fig. 1C)^41^. Mono Q is a strong anion exchange column with advantage of adsorption the negative net charge of endotoxins, have been extensively used for endotoxins adsorption and removal^42^. The level of endotoxin contamination in this rLipL32 fraction was significantly low (Fig. S4). To reduce the LPS contamination even further, rLipL32WT and its variants were subjected to polymyxin B resin. Endotoxin contamination was notably decreased after purified by mono Q ion exchanged column and polymyxin B resin (<0.1EU/mg; p < 0.001) (Fig. S4). The molecular mass of rLipL32 and its variants were assay by using Superdex 200 gel filtration column (Fig. 1C). The structure stability and integrity of rLipL32 and its relative variants were measured by CD and the results indicated that the central β-sandwich domain of rLipL32 was stable when deletion the domains mentioned above (Fig. 1D). Further identification the structure and function integrity of rLipL32 deletion variants, the Ca^2+^ binding ability was measured by stains-all assay and the result represented that the deletion variants of rLipL32 could bind stains-all and the J band at 650 nm were observed (Fig. 1E). The Ca^2+^ binding domain of rLipL32 is located at the poly D motif within the central β-sandwich domain of rLipL32^25^ and the results indicated that the deletion of the three domains of rLipL32 maintained the Ca^2+^ binding ability. These results also showed that the structure integrated and Ca^2+^ binding ability of rLipL32 protein when deletion of possible rTLR2 binding domains. Therefore, the variants of the affinity to rTLR2 of rLipL32 truncated variants are resulting from the deletion of the vital domains involved in LipL32-TLR2 complex formation.

### LipL32 co-localizes with TLR2 on HEK293 cells

**Figure 2:**
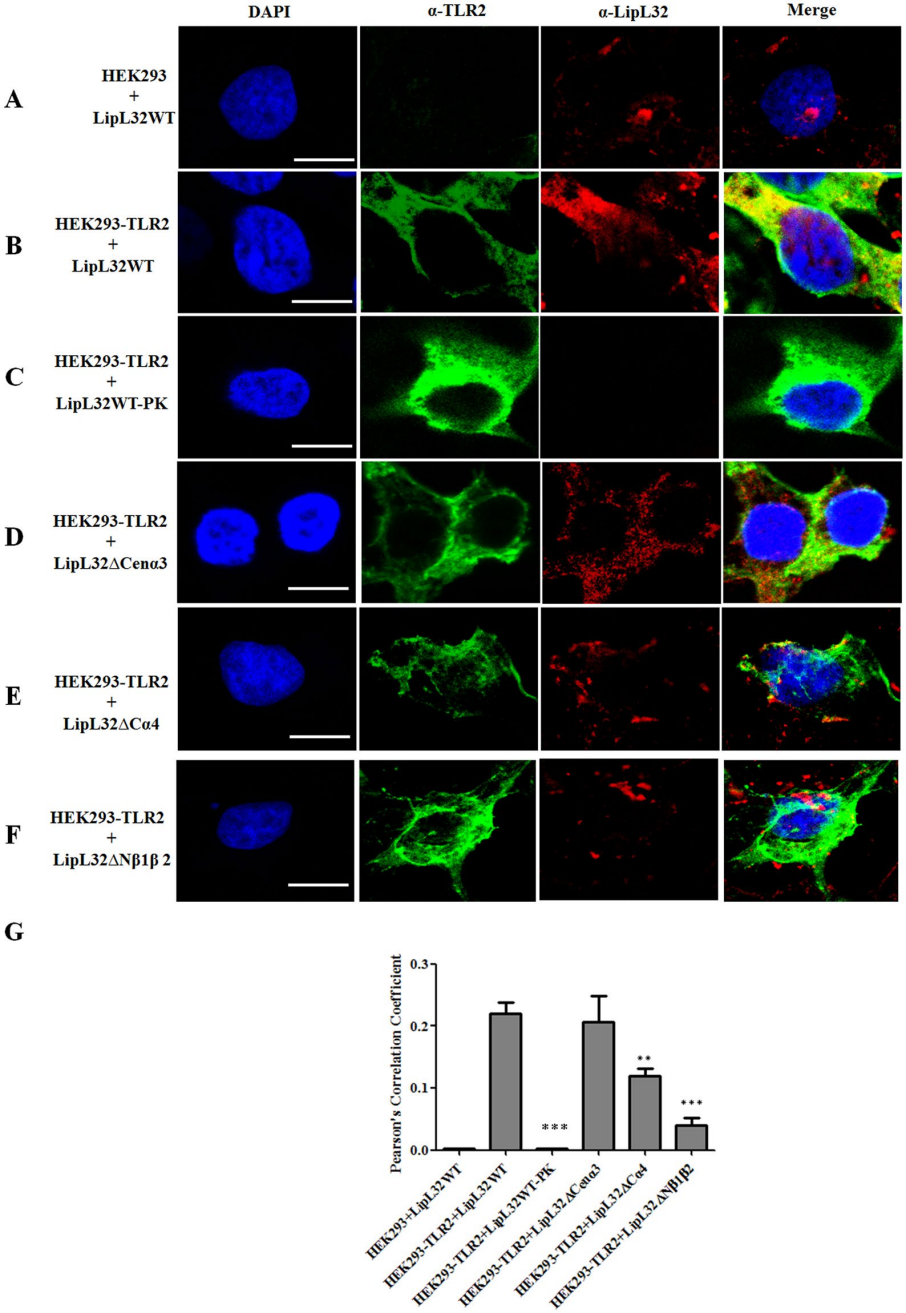
Co-localization of LipL32 and TLR2. The confocal microscopy was used to observe the co-localization of LipL32 and TLR2 on HEK293-TLR2 cell. The purified LipL32 variants were incubated with HEK293-TLR2 cell as described in Materials and Methods and observed by confocal microscopy. TLR2 was stained by Alexa488 (green) and LipL32 was stained by Alexa594 (red). The yellow color indicated that the two proteins were co-localized on HEK293-TLR2 cell. (**A**) HEK293 cell incubated with LipL32WT. (**B**) HEK293-TLR2 cell incubated with LipL32WT. (**C**) HEK293-TLR2 cell incubated with LipL32WT treated with proteinase K (PK). (**D**) HEK293-TLR2 cell incubated with LipL32ΔCenα3. (**E**) HEK293-TLR2 cell incubated with LipL32ΔCα4. (**F**) HEK293-TLR2 cell incubated with LipL32ΔNβ1β2. (**G**) Statistic analysis of the co-localization of the LipL32 and TLR2 on HEK293-TLR2 cell. Pearson’s correlation was used to calculate the overlap between image pairs. All conditions are repeated at least three independent experiments. Scale bar, 7.5 μm; **p < 0.01; ***p < 0.001.

In the infection of host cell with *Leptospira*, outer membrane proteins of *Leptospira* are the first line to contact the host cell and interact with the cell membrane components. LipL32 is the most abundant outer membrane lipoprotein and occupied about 75% of the total outer membrane protein profile^43^. Previous studies have illustrated that LipL32 binds to different components of the ECM, including fibronectin, collagen, and laminin, respectively^9, 22^. Besides ECM molecules, LipL32 also interacts with innate immune components, such as TLR2, and induces inflammatory responses^25, 26^. However, the domains of LipL32 responsible for interacting with TLR2 are unclear. Therefore, confocal microscopy was used to observe the co-localization of truncated rLipL32 variants with the TLR2 receptor on the HEK293 cell surface (Fig. 2). Purified rLipL32 variants were used to incubate with HEK293 or HEK293-TLR2 cells for 4 h, after which the cells were washed three times with PBS buffer to remove non-interacting rLipL32 proteins and fixed for confocal microscopy analysis. rLipL32 and rTLR2 proteins were stained with rabbit polyclonal anti-LipL32 and mouse monoclonal anti-V5 primary antibodies fellow by Alexa594 (red) conjugated anti-rabbit and Alexa488 (green) conjugated anti-mouse secondary antibodies, respectively. HEK293 cells lacking the expression of rTLR2 protein were used as negative control and very little or no fluorescence of Alexa 488 signal (Fig. 2A). As rLipL32 proteins could bind to the cell surface of these HEK293 cells as well, it seems that rLipL32 proteins associate with cell surface ECM molecules^9^. In rTLR2 expressing HEK293 cells, most of rLipL32WT proteins co-localized with TLR2 receptors (Fig. 2B). This provides direct evidence that the rTLR2 receptor and rLipL32 proteins were co-localize on the cell surface (Fig. 2B, yellow color). Further confirming the rLipL32 protein co-localized with rTLR2, the rLipL32 protein was treated with proteinase K (PK) for 30 min before incubating with HEK293-TLR2 cell and the results indicated the reducing the ability to bind rTLR2 (Fig. 2C). To compare different truncations of rLipL32 in their interaction with the rTLR2 receptor, HEK293-TLR2 cells were incubated with rLipL32 truncation variants to evaluate co-localization phenomenon. Interestingly, although most of the ΔNβ1β2 variants of rLipL32 were bound to cell surface, they did not co-localize with TLR2 (Fig. 2F). In contrast, the rLipL32ΔCenα3 variant showed similar behavior as compared to rLipL32WT (Fig. 2D). Pearson’s Correlation was further used to elucidate the co-localization between rTLR2 and rLipL32 truncated variants. rLipL32ΔCα4 (p < 0.01) and rLipL32ΔNβ1β2 (p < 0.001) significantly decreased the co-localization to TLR2 (Fig. 1G). The results suggested that the N- and C-termini of rLipL32 play a critical role in rTLR2 interaction.

### Inflammatory responses induced by the leptospiral outer membrane protein LipL32

**Figure 3:**
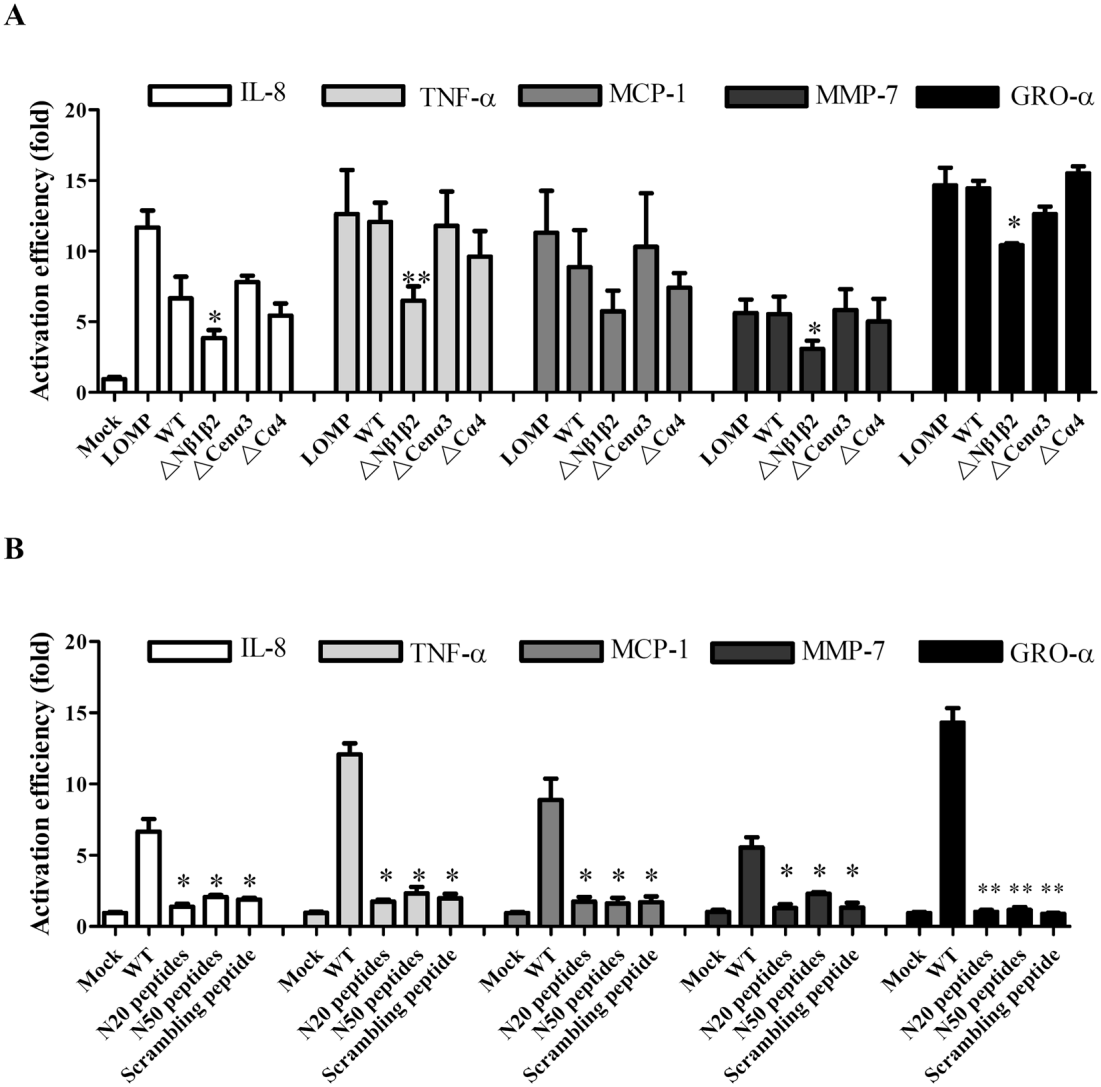
Stimulation of inflammatory responses by LipL32WT and its variants. (**A**) LipL32WT and its variants were used to stimulate the expression of *IL-8* (white bar), *TNF-α* (light gray bar), *MCP-1* (gray bar), *MMP7* (dark gray bar), and *GRO-α* (black bar) on HEK293-TLR2 cell. LOMP, *Leptospira* outer membrane protein extraction; WT, wild-type LipL32 protein; ΔNβ1β2, deletion of β1β2 domains of LipL32; ΔCenα3, deletion of central α3 domain of LipL32; ΔCα4, deletion of C-terminal α4 domain of LipL32. (**B**) The synthesized peptides were used to stimulate the expression of *IL-8* (white bar), *TNF-α* (light gray bar), *MCP-1* (gray bar), *MMP7* (dark gray bar), and *GRO-α* (black bar) on HEK293-TLR2 cell. N20 peptide (1–20 residues of LipL32); N50 peptide (1–50 residues of LipL32); C25 peptide (248–272 residues of LipL32), and a scrambling peptide with the sequence of VSLEGLTKVLAGSKFSESPILIKTVCDMILGASFANPALKISTASTLFTG. *p < 0.05; **p < 0.01.

Recognition of bacterial components by host TLRs initiates signaling cascades that stimulate nuclear transcription factor κB (NF-κB) and mitogen-activated protein kinases (MAPKs), and induces the expression of chemokines and cytokines^8, 25–27^. The expression levels of *hCXCL8/IL8* (IL-8), *hCCL2/MCP-1* (MCP-1), *hTNF-α* (TNF-α), *hMMP7* (MMP-7), *hCXCL1/GRO-α* (GRO-α), and *osteopontin/SPP1* (SPP1)^8, 27^ were measured to investigate TLR2 activation by its ligands (Fig. 3). Two hours after adding 2.5 μg/ml stimulating agents (including leptospiral outer membrane protein extraction [LOMP], WT, ΔNβ1β2, ΔCenα3, and ΔCα4 of rLipL32) to HEK293-TLR2 cells, the mRNA levels of *IL8*, *MCP-1*, *hTNF-α*, *hMMP7*, and *GRO-α* were changed by addition of the truncated rLipL32 proteins as compared to WT. However, these virulence factors could not stimulate the expression of SPP1. LOMP stimulated the expression of IL-8, MCP-1, TNF-α, MMP-7, and GRO-α and considered as a positive control whereas the PBS buffer acted as mock control (Fig. 3)^25^. To test the effect of a His_6_-tag on rLipL32, His_6_-tagged and un-tagged rLipL32 were used to induce the inflammatory responses and the results indicated that both forms of rLipL32 stimulated similar level of IL-8, MCP-1, TNF-α, MMP-7, GRO-α, and SPP1 (Fig. S5A). The two forms of rLipL32 showed no significant difference in their ability to induce inflammatory responses. Thus, the presence or absence of His_6_-tag on rLipL32 has no obvious effects on their ability to induce inflammatory responses. We next examined whether downstream signaling was directly induced by rLipL32 proteins, therefore, and the proteins were treated with heat (100 °C, 30 min) and proteinase K (20 µg/ml at 63 °C for 18 h). The results showed that rLipL32 protein treated with heat and proteinase K had a significantly lower level of inflammatory induction compared to that of WT (Fig. S5B; p < 0.01). In addition, the expression of IL-8, TNF-α, MCP-1, MMP-7, and GRO-α were measured by addition of rLipL32WT and its variants, including ΔNβ1β2, ΔCenα3, and ΔCα4. rLipL32ΔNβ1β2 significantly decreased the expression levels of IL-8 (p < 0.05), TNF-α (p < 0.01), MMP-7 (p < 0.05), and GRO-α (p < 0.05) compared to that of rLipL32WT, whereas ΔCenα3, and ΔCα4 variants showed no significant difference in stimulating the expression of IL-8, TNF-α, MCP-1, MMP-7, and GRO-α as compared to hat of rLipL32WT (Fig. 3A). These results indicate that the N terminal β sheet domains of rLipL32 are a potential candidate for induction of the inflammatory responses through TLR2 signaling.

To confirm the N terminal β sheet as the binding domain, the synthesized peptide including N20 peptide (1–20 residues of LipL32), N50 peptide (1–50 residues of LipL32), C25 peptide (248–272 residues of LipL32), and a scrambled peptide with the sequence of VSLEGLTKVLAGSKFSESPILIKTVCDMILGASFANPALKISTASTLFTG, were used to induce inflammatory responses. With the exception of the N50 peptide, all these synthesized peptides stimulated less than 2-fold inflammatory responses compared to that stimulated by the mock control (Fig. 2B). The N50 peptide stimulated the expression of IL8, TNF-α, MMP7 by 2.1-, 2.3-, and 2.3-fold, respectively. The synthesized peptides revealed significantly decreased the stimulation level of IL-8 as compared to WT (Fig. 2E–H; p < 0.05). Thus, the stimulation of inflammatory responses provoked by the N50 peptide indicates that the binding domain is located within the N terminal region.

### The interaction between LipL32 and TLR2 as determined by ELISA

**Figure 4:**
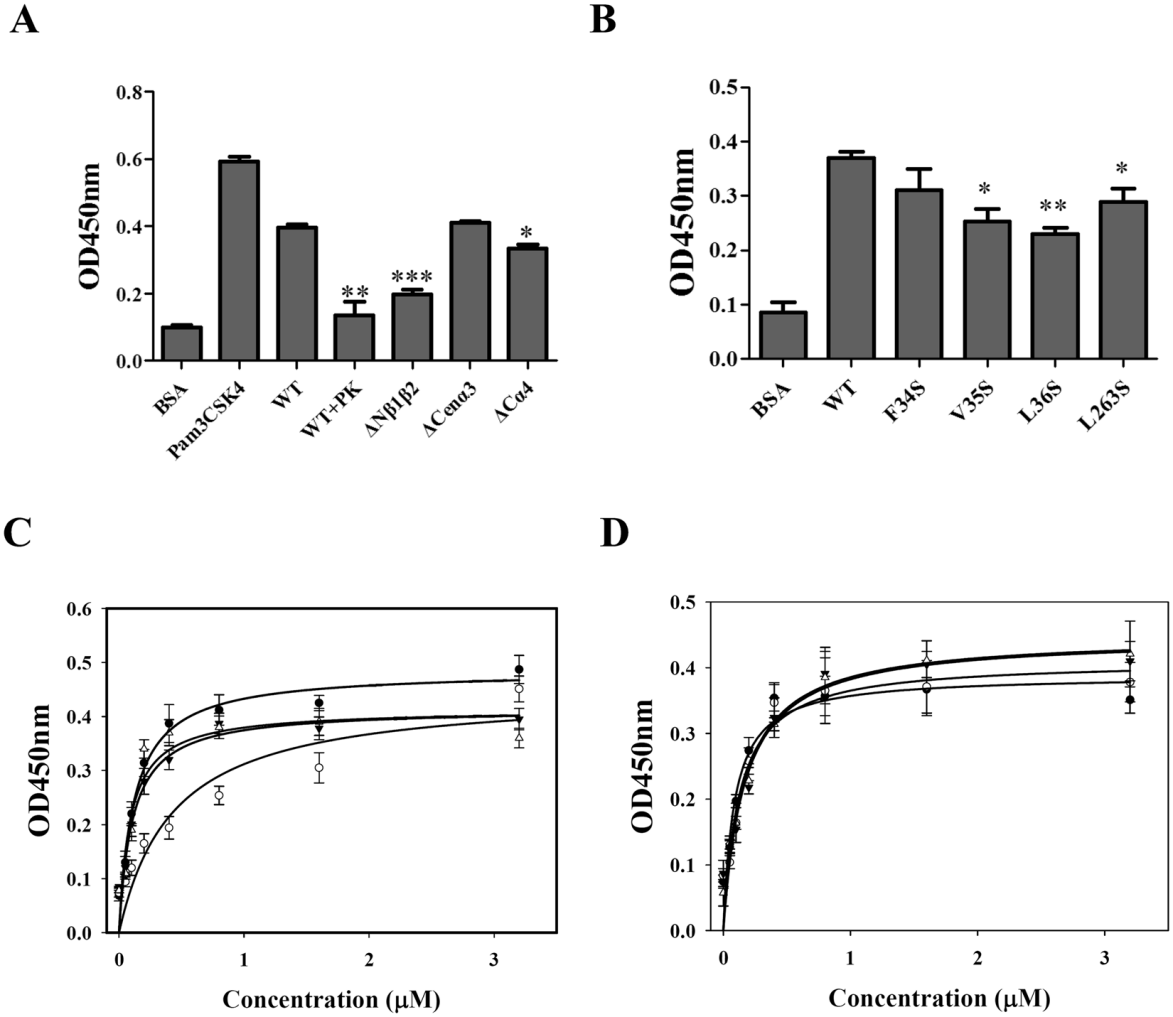
The ELISA assay of the interaction between TLR2 protein and LipL32 variants. (**A**) The interaction of TLR2 protein and LipL32 truncated variants. The protein concentration of LipL32 and TLR2 protein were 1 µM. BSA and Pam_3_CSK_4_ were served as negative and positive controls. WT + PK indicated the LipL32WT pretreated with proteinase K for ELISA assay. (**B**) The interaction of TLR2 protein and LipL32 point mutation variants. (**C**) The dose dependent ELISA of the interaction between LipL32ΔCenα3 variant and TLR2 protein. ○, LipL32WT in the presence of 1 µM Ca^2+^; ●, LipL32WT in the presence of 1 µM EGTA; ▼, LipL32ΔCenα3 in the presence of 1 µM Ca^2+^; △, LipL32ΔCenα3 in the presence of 1 µM EGTA. (**D**) The dose dependent ELISA of the interaction between LipL32 point mutation variants and TLR2. ○, D195A variant in the presence of 1 µM Ca^2+^; ●, D195A variant in the presence of 1 µM EGTA; ▼, D196A variant in the presence of 1 µM Ca^2+^; Δ, D196A in the presence of 1 µM EGTA. *p < 0.05; **p < 0.01; ***p < 0.001.

Previous studies indicated that LipL32 directly interacts with TLR2, and we further investigated the molecular basis of interaction between rLipL32 and rTLR2^25^. Using ELISA, we first analyzed the binding of rTLR2 protein to its ligands, including Lipoteichoic acid (LTA), Pam_3_CSK_4_, and peptidoglycan (PGN), and these results indicating that the rTLR2 protein from our system was suitable for ligand binding assays (Fig. S3A)^25^. To identify the active components involved in the interaction between rLipL32 and rTLR2, the binding efficiency of rLipL32WT and its variants, including rLipL32ΔNβ1β2, rLipL32ΔCenα3, and rLipL32ΔCα4, in associating with rTLR2 were determined (Fig. 4A). rLipL32WT pretreated with proteinase K significantly decreased the affinity to rTLR2 (Fig. 4A, p < 0.01). rLipL32ΔNβ1β2 (p < 0.001) and rLipL32ΔCα4 (p < 0.05) had significantly decreased binding efficiency and rLipL32ΔCenα3 showed no significant difference in binding efficiency, as compared to that of rLipL32WT (Fig. 4A). This result indicates that the N terminal β1β2 and C terminal α4 domains may be involved in interaction with rTLR2. We next examined the residues within the two domains of rLipL32 are involved in the interaction with rTLR2. Previous studies indicated that rTLR2 binding proteins may utilize hydrophobic residues to interact with the LRR domain of rTLR2^36, 38, 39^. In addition, the molecular docking analysis for Model III indicated the residues with the nearest distance to rTLR2, including Phe^34^, Val^35^, Leu^36^, and Leu^263^; these residues were mutated to Serine and the affinity between mutant variants of rLipL32 and rTLR2 were measured (Fig. S1). The V35S and L263S variants of rLipL32 had significantly decreased affinity to rTLR2 compared to that of rLipL32WT (p < 0.05); the L36S variant showed significantly decreased the affinity to rTLR2 compared to rLipL32WT (p < 0.01) (Fig. 4B). These results further confirmed that the N terminal β1β2 and C terminal α4 domains are involved in interacting with rTLR2 and the residues Val^35^, Leu^36^, and Leu^263^ possibly involved in the interaction interface of rLipL32-rTLR2 complex. It also suggests that rLipL32 uses hydrophobic residues to interact with rTLR2.

The role of Ca^2+^ in regulating in the interaction between rLipL32 and rTLR2 was shown by dose-dependent ELISA, and the binding affinity of rLipL32 variants to rTLR2 was measured (Fig. 4C). The dissociation constant (*K*_*d*_) was calculated as 0.13 ± 0.02 µM from Eq. 1 for the binding of rLipL32WT to rTLR2 proteins (Fig. 4C). A previous study confirmed that Ca^2+^ regulates the interaction of rTLR2 and rLipL32 as well as poly-D motif variants of rLipL32, which reduce regulation ability^25^. The role of Ca^2+^ ions was further investigated in these deletion variants of rLipL32. In the presence of ethylene glycol tetraacetic acid (EGTA), the affinity of rLipL32WT to rTLR2 was decreased to 0.45 ± 0.12 µM as compared to that in the presence of Ca^2+^ ions, demonstrating that sequestration of Ca^2+^ ion reduced the binding affinity of rLipL32 to rTLR2 proteins (Fig. 4C)^25^. This also demonstrates that rLipL32 and rTLR2 interact in a Ca^2+^ dependence manner. To test the role of Ca^2+^ in regulating the rLipL32-rTLR2 interaction, the central α3 domain of rLipL32 was removed (LipL32ΔCenα3 variant), and the affinity of rLipL32 to rTLR2 was measured in the absence and presence of Ca^2+^ (Fig. 4C). Interestingly, after the deletion of the central α3 domain, this variant showed similar *K*_*d*_ values in either the absence or presence of Ca^2+^ ions, indicating that rLipL32ΔCenα3 variant might shifts from Ca^2+^ dependent to Ca^2+^ independent of affinity for rTLR2 protein. According to the crystal structure of LipL32 in the absence and presence of Ca^2+^ ions, the conformational change that occurs in the Cenα3 domain suggests that this domain is the Ca^2+^ regulation motif. In addition, Cluspro predicted that the Cenα3 domain was close to the TLR2 protein and that this domain may be involved in interacting with TLR2. However, the affinity of rTLR2 to rLipL32ΔCenα3 showed no significant difference compared to that in rLipL32WT (Fig. 4A). Therefore, the Cenα3 domain may instead play a role as an insulator that interferes with the interaction of rLipL32 and rTLR2 in the absence of Ca^2+^.

We next tested the importance of Cenα3 for the rLipL32-rTLR2 interaction. Two residues, D195 and D196, within Cenα3 domain were selected and mutated to Ala for affinity analysis. In dose- dependent ELISA, the *K*_*d*_ values of D195A and D196A were calculated as 0.10 ± 0.03 µM and 0.16 ± 0.04 µM, respectively, in the presence of Ca^2+^ (Fig. 4D). In the treatment of EGTA, the *K*_*d*_ values of D195A and D196A were calculated as 0.12 ± 0.04 and 0.16 ± 0.03 µM, respectively. Thus, the binding affinity of these two variants showed similar behavior in their interaction with rTLR2, further confirming the role of the Cenα3 domain. The charged residues, D195 and D196 within the Cenα3 domain may interfere with the interaction between rLipL32 and rTLR2; replacing both of the charged residues to Ala altered the ability of Ca^2+^ to regulate of the interaction.

### Single-molecule interaction between LipL32 and TLR2 as determined by AFM

**Figure 5:**
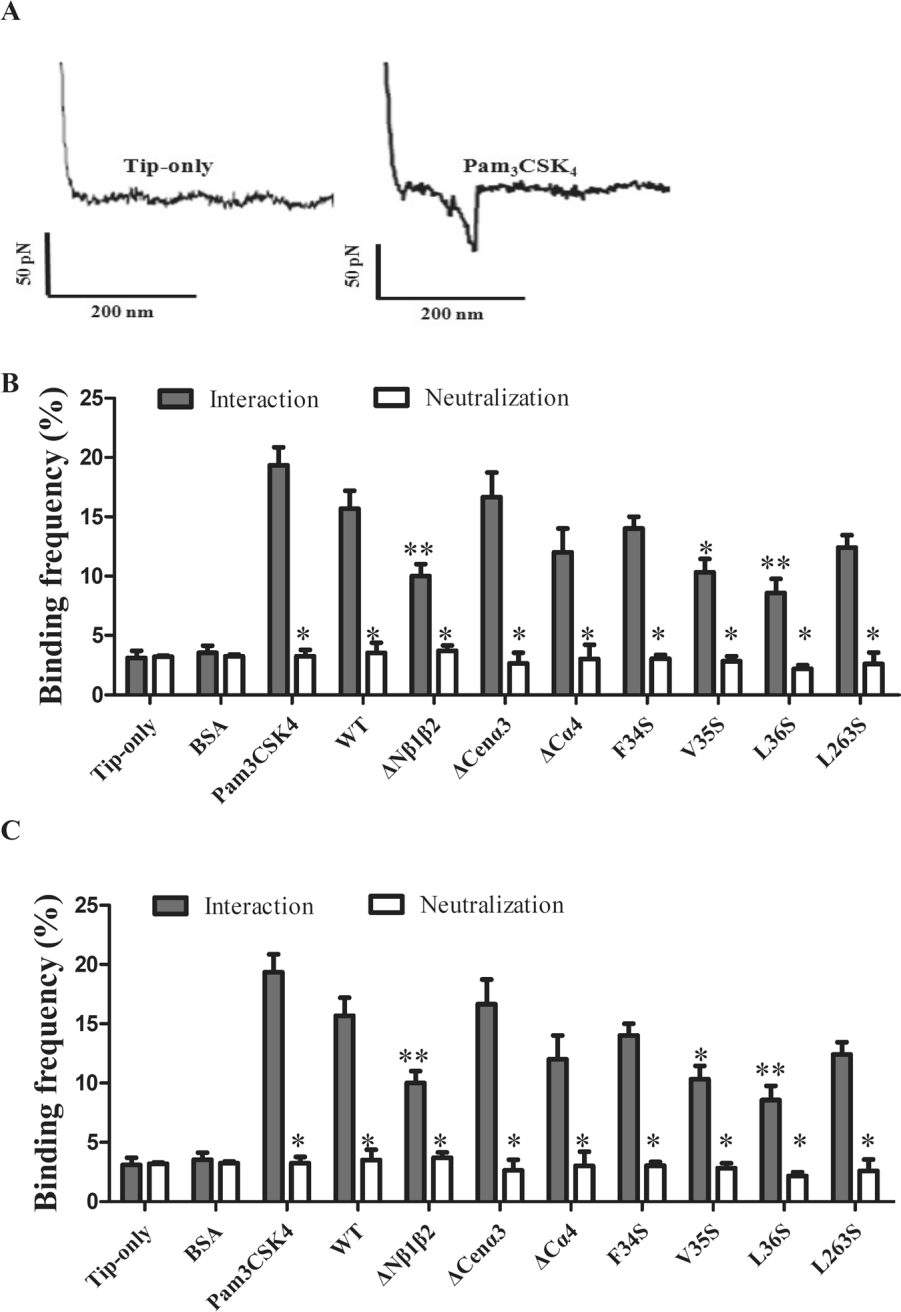
The AFM measurement of the interaction between TLR2 and LipL32 variants. The maximum distribution force was calculated and determined the interaction force between different fragments of LipL32 and TLR2 protein. (**A**) The force-distance curves of AFM measurement. The tip-only (left panel, mock control) and Pam_3_CSK_4_ (right panel, positive controls) modified AFM tip were used to analyze the interaction between tip and TLR2 modified mica surface. (**B**) The interaction force between LipL32 variants and TLR2 protein. The different truncated and mutated variants of LipL32 were modified on AFM tip and the interaction forces to TLR2 were therefore measured. The anti-TLR2 antibody was used to neutralize the interaction between LipL32 and TLR2 and the interaction forces were calculated. (**C**) The binding frequency between LipL32 truncated and mutated variants and TLR2 protein. The binding frequency was calculated from the interaction of different LipL32 variants and TLR2 protein. The anti-TLR2 antibody was used to neutralize the interaction between LipL32 and TLR2 and the binding frequency were calculated. Gray bars, LipL32-TLR2 interaction; white bars, anti-TLR2 antibody neutralization. *p < 0.05; **p < 0.01; ***p < 0.001.

To further investigate the mechanisms behind rLipL32-rTLR2 interaction, a powerful tool for investigation of protein-protein interaction, AFM, was employed to determine the role of the active components in the rLipL32-rTLR2 interaction. rTLR2 was first deposited through covalent modification on a mica surface, while the tip was modified with rLipL32 or its variants to investigate binding interactions. The force-distance curves were recorded and tip-only and Pam_3_CSK_4_ were used as mock and positive controls, respectively (Fig. 5A). The AFM tip modified with Pam_3_CSK_4_ was not only used as a positive control for interaction between rTLR2 and its ligand but also to confirm the activity of rTLR2 on a mica surface. The interaction force in the force-distance curve between rTLR2 and Pam_3_CSK_4_ was measured and the maximum distribution force was calculated as 67.4 ± 2.4 pN (Fig. 5A). When neutralizing by anti-TLR2 antibody, the maximum distribution force was significantly decreased to 23.1 ± 1.8 pN (p < 0.05). This result indicated that the interaction directly contributed from the Pam_3_CSK_4_-TLR2 interaction. The force-distance curve was also used to determine the rLipL32WT-rTLR2 interaction, and the maximum distribution force was calculated as 55.3 ± 1.5 pN^25, 26^ (Fig. 5B). We next examined the interaction forces between rLipL32 truncated variants and rTLR2. rLipL32ΔNβ1β2 and rLipL32ΔCα4 showed significantly decreased interaction forces between rLipL32 and rTLR2 (p < 0.001) (Fig. 5B). In contrast, ΔCenα3 of rLipL32 revealed no significant difference in interaction force between rLipL32 and rTLR2. The result is consistent with the ELISA assays, which suggested that the binding domains were located at the N- and C- terminus. In addition, an anti-TLR2 antibody was used to neutralize the interaction between rLipL32 variants and rTLR2 and binding interactions were measured by AFM. These results showed that addition of anti-TLR2 antibodies significantly decreased the interaction force (p < 0.05) compared to that in the control (Fig. 5B). In addition, the binding frequency between rLipL32 variants and rTLR2 were determined indicating that rLipL32ΔNβ1β2 significantly diminished the binding frequency between rLipL32 and rTLR2 (p < 0.001) (Fig. 5C). In contrast, rLipL32ΔCenα3 and rLipL32ΔCα4 showed no significant difference in binding frequency. Together, these results suggest that the N-terminal β1β2 and C-terminal α4 domains play a major role in promoting interaction between rLipL32 and rTLR2.

To further elucidate the interacting domain, point mutations of rLipL32, including F34S, V35S, L36S, and L263S variants, were used and interaction forces were determined from AFM force-distance curves. The results revealed that V35S (p < 0.01), L36S (p < 0.01), and L263S (p < 0.05) showed significantly reduced interaction force compared to WT, whereas F34S showed no significant difference compared to WT measurements (Fig. 5B). Additionally, when using anti-TLR2 antibody to neutralize the LipL32-TLR2 interaction, the interaction forces and binding frequency of rLipL32-rTLR2 interaction were significantly decreased (p < 0.05) as compared to those without antibody treatments (Fig. 5B,C).

### Peptides inhibit the interaction between LipL32 and TLR2

**Figure 6:**
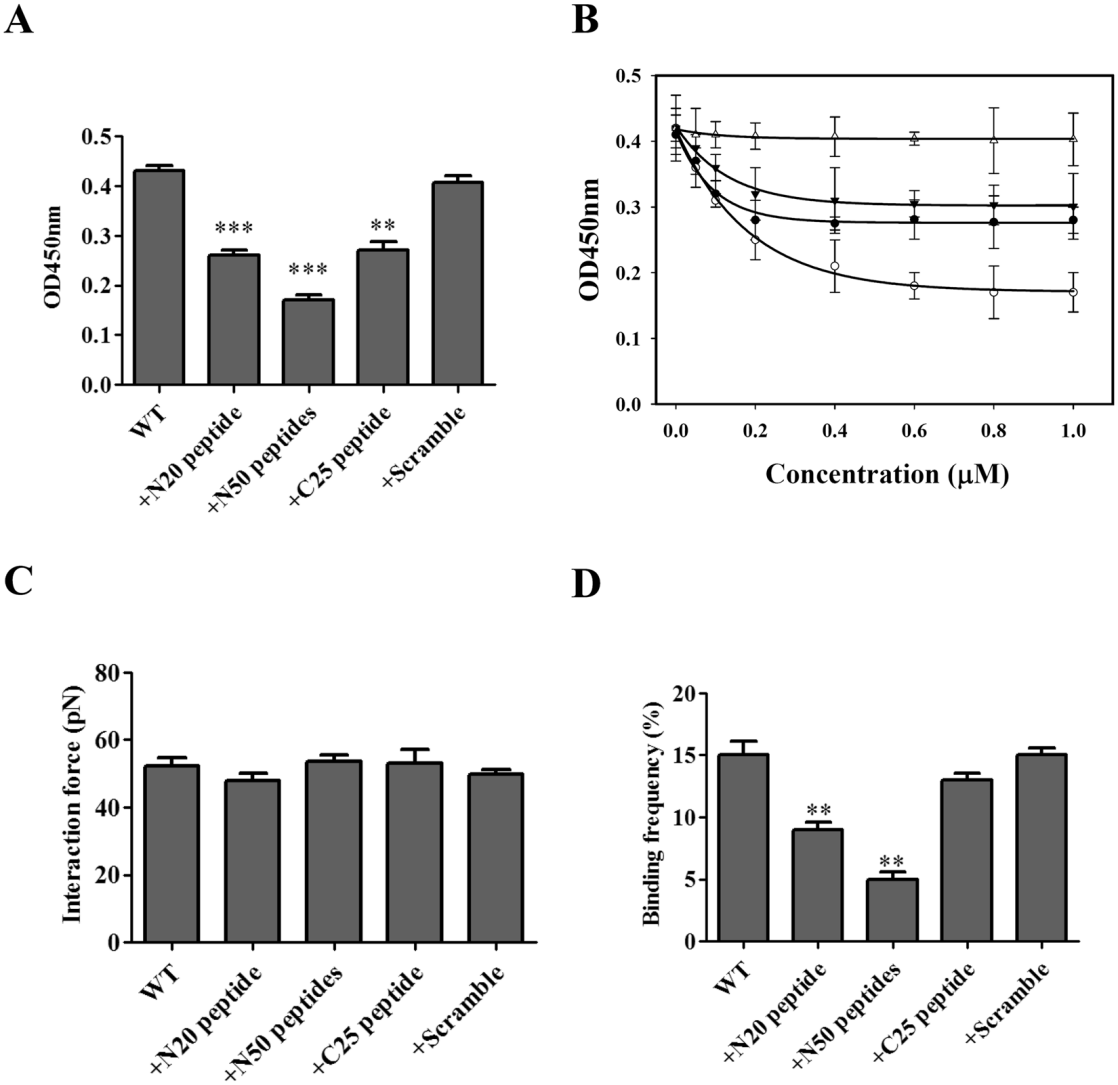
The inhibition of LipL32-TLR2 interaction by synthesized peptides. One μM synthesized peptides including N20, N50, C25, and scramble peptides were used to inhibit the interaction between LipL32WT and TLR2 protein. (**A**) The inhibition of LipL32 and TLR2 interaction by synthesized peptides as measured by ELISA. (**B**) Dose-dependence ELISA assay of the peptides inhibition. (**C**) The inhibition of LipL32 and TLR2 interaction by synthesized peptides as measured by AFM force-distance curves. (**D**) The AFM binding frequency assay of peptides inhibition the LipL32 and TLR2 interaction. *p < 0.05; **p < 0.01; ***p < 0.001.

We next examined the effect of synthesized peptides on the rLipL32-rTLR2 interaction. Synthesized peptides were based on the sequence of LipL32 and used to inhibit the rLipL32WT-rTLR2 interaction (Fig. 6). ELISA was used to measure the interaction of rLipL32-rTLR2, and 1 μM synthesized peptides including, N20, N50, C25, and scrambled peptides, were used to compete with the interaction between rLipL32WT and rTLR2. The results showed that N20 (p < 0.001), N50 (p < 0.001), and C25 (p < 0.01) significantly inhibited the rLipL32-rTLR2 interaction (Fig. 6A). In addition, dose dependent ELISA was used to measure the half maximum inhibition (IC_50_) of the rLipL32-rTLR2 interaction. The IC_50_ of N20, N50, C25, and scrambled peptides were calculated as 0.09 ± 0.05 μM, 0.07 ± 0.04 μM, 0.14 ± 0.01 μM, and 13.9 ± 1.2 μM, respectively (Fig. 6B). These results suggest the two terminal peptides are rTLR2 binding candidates that compete in binding of rTLR2 to rLipL32. In addition, AFM was used to measure rLipL32WT-rTLR2 interaction in the presence of competing synthesized peptides. Interaction forces were similar in the presence of the synthesized peptides. However, the binding frequency of the rLipL32-rTLR2 interaction was significantly decreased in the presence of N20 and N50 peptides (p < 0.01). This result suggests that the N20 and N50 synthesized peptides play a major role in inhibiting the rLipL32-rTLR2 interaction. The inhibition mediated by these synthesized peptides also verifies that the N terminal β1β2 domains are critical for interacting with rTLR2.

## Discussion

The TLR family comprises pattern recognition receptors and their ligands originate from microbes and contain multiple conserved patterns that can be recognized by the TLR LRR domain^34, 44^. TLR2 protein forms homo- or hetero- dimeric structure with TLR1 or TLR6 to specifically recognize a wide range of ligands. Previous study indicated that the HEK293 cell transfected with pUNO-TLR2 could stimulated inflammatory responses by adding the *Leptospira* LipL32 indicated that TLR2 homodimer were responsible for LipL32 stimulation^27^. Several conserved patterns from bacteria have been identified as TLR2 ligands, including lipidation molecules (e.g. lipoprotein, lipopeptide, PGN, LTA, etc.)^35^, leucine-rich repeat family proteins (BspA)^38^, proteins with TLR2 binding sequence (PorB)^45^, and proteins with the specific structures for TLR2 binding (enterotoxins)^39^. Crystal structure of the TLR2/1 complex associated with the ligand, Pam_3_CSK_4_, revealed that the lipid molecule of the lipoprotein supported the binding to TLR2^35^. Previous studies showed that the LipL32 protein is modified with lipid molecules at the N-terminus^16–18^. Interesting, other study showed that non-lipidated polypeptides lack stimulation activity via TLR2 pathway^46^. However, an increasing studies revealed that polypeptides ligands could interact to TLR2 and stimulate or inhibit the inflammatory responses through TLR2 pathway^36, 38, 39, 45^.

In our study, deletion of the signal peptide of rLipL32 did not prevent binding to rTLR2, and it was still able to stimulate inflammatory responses, suggesting that other domains of rLipL32 are responsible for interacting with rTLR2^25, 26, 28^. The mature form of rLipL32 protein is a lipoprotein in which the first 20 residues are removed. Therefore, the rLipL32ΔN20 construct is considered as wild-type rLipL32 protein for comparison with other truncated variants. Crystal structure of rLipL32, a jellyroll conformation without the LRR domain in its structure^19–21^, appears unlikely to use the LRR domain to interact with rTLR2^38^. In addition, structure alignment showed that the structure of rLipL32 is substantially different from that of enterotoxin B^39^. Moreover, sequence alignment of rLipL32 with and other TLR2 binding proteins such as BspA, PorB, and LcrV suggested that the homology of these proteins are low (<10%), and TLR2 binding motifs from BspA, PorB, and LcrV were not found in LipL32. These results indicate that rLipL32 is a unique protein with specific sequence and structure that different from the known TLR2 ligands. As a novel TLR2 ligand, rLipL32 is suggested to utilize its distinctive structure and mechanism to interact with TLR2.

The endotoxin contamination from the recombinant protein interfere the intrinsic inflammatory properties of the innate immunity system. To overcome this problem, the endotoxin locked expression system, ClearColi^TM^ BL21(DE3) was used to produced endotoxin free recombinant proteins^47^. The yield of protein expression are similar to that expressed in BL21(DE3) competent cell. In addition, the anion-exchange chromatography (Mono-Q) and polymyxin B resin were further used to remove contaminating endotoxin from the purified rLipL32 variants^41^. In the LAL assay, it was observed that the endotoxin was significantly removed. In order to prevent the interference of the resident endotoxin, the HEK293-TLR2 cell naturally not expressing TLR4 was used to measure the inflammatory responses. Therefore, the responses from the stimulation of HEK293-TLR2 were directly from the rLipL32 and its variants.

The synthesized peptides derived from LipL32 were used to interact with rTLR2. However, the affinity of synthesized peptides to rTLR2 was low as determined in ELISA and AFM analysis. Previous study in BspA protein revealed that the synthesis truncated peptides (LRR domains) could induce responses to interact with rTLR2. We speculated that the synthesis peptides derived from LipL32 lacking suitable conformation to interact with rTLR2. Interestingly, the synthesized peptides, N20 and N50 peptides were used to compete the interaction between rLipL32 and rTLR2 (Fig. 6). However, the C25 peptide failed to compete this interaction indicated that C25 peptide is not the major domain to interact with rTLR2. The C25 domain of LipL32 is suggested as the assisted role in rLipL32-rTLR2 interaction.

Furthermore, the residues at N- and C-termini of rLipL32 are investigated to find out the residues responsible for interacting to rTLR2. In the predicted complex, several resides of rLipL32 with nearest distance to rTLR2 were replace to Serine residues and the mutation variants were purified to measure the affinity to rTLR2. Interestingly, our prediction rLipL32-rTLR2 complex and experimental data also suggest that three hydrophobic residues, including Val^35^, Leu^36^, and Leu^263^, are involved in rLipL32-rTLR2 complex interface. Similarly, protein complex of SSL3-TLR2 was solved and the residues involved in complex formation were proposed^36^. The interface of the SSL3-TLR2 complex is a hydrophobic region which contains Phe^156^, Phe^158^, Leu^160^, and Pro^194^ of SSL3 as well as Phe^349^, Leu^350^, Gln^375^, Tyr^376^, and Asn^379^ of TLR2. In addition, the pentameric B subunit of type IIb *E. coli* enterotoxin (LT-IIb-B_5_) also suggests its hydrophobic upper-pore region directly binds to TLR2 and four residues, including Met^69^, Ala^70^, Leu^73^, and Ser^74^ are responsible for TLR2-binding. Therefore, protein ligands for TLR2 with the hydrophobic patch may play vital roles in interacting to TLR2.

**Figure 7:**
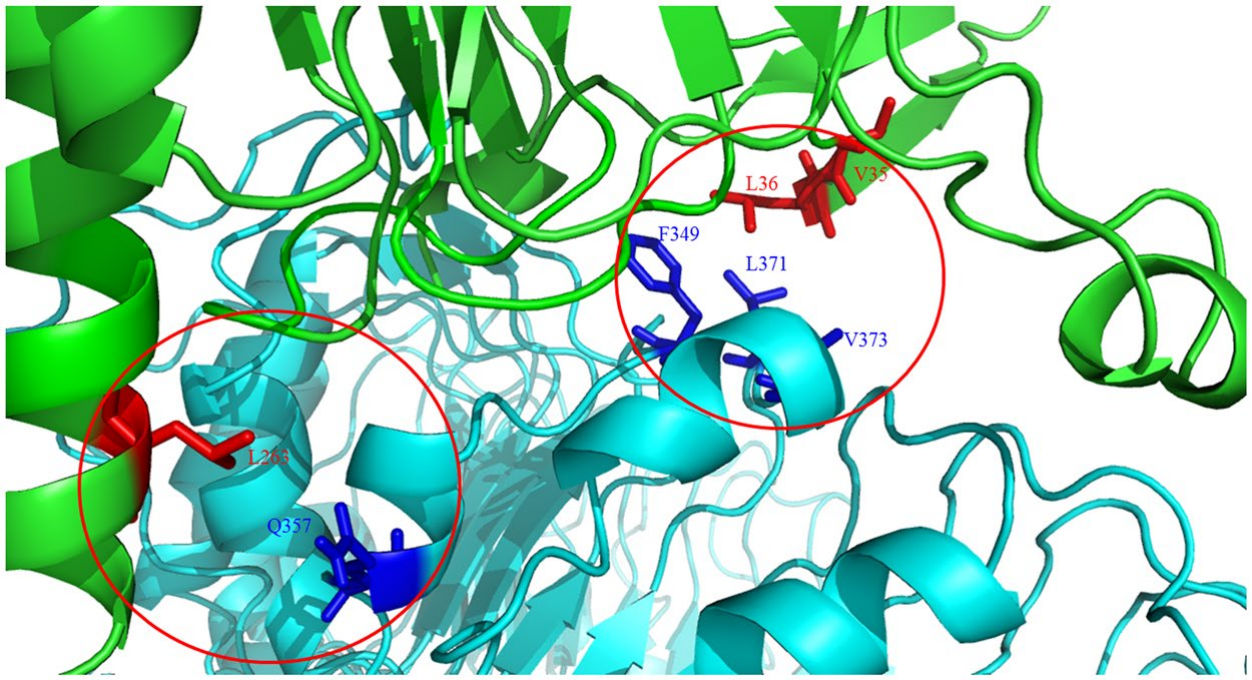
The proposed interaction interface of LipL32-TLR2 complex. The protein docking prediction of LipL32-TLR2 complex shows the N- and C-termini of LipL32 play vital roles of complex formation. The proposed binding interface of the LipL32-TLR2 complex indicates the residues of the LipL32 involving in complex formation (red color) and the predicted nearest distance to residues of TLR2 (blue color). Green, LipL32; cyan, TLR2.

In summary, both computational prediction of the complex and experimental data suggest that rLipL32 interacts with rTLR2 through both N and C termini. The predicted model suggested that the N-terminus is inserted into the LRR domain of rTLR2 and C-terminus assists in the binding of LipL32 to TLR2. The two domains of LipL32 form a plier-like structure to interact with the rTLR2 LRR domain. Val^35^, Leu^36^, and Leu^263^ at N and C terminus play pivotal roles in interaction to TLR2, respectively. The corresponding binding residues of rTLR2 were predicted using nearest distance prediction and the results show the hydrophobic interaction of Phe^349^ and Leu^371^ of rTLR2 to Leu^36^ of rLipL32; Leu^350^, Gln^357^ of rTLR2 to Leu^263^ of rLipL32, respectively (Fig. 7). Interestingly, the binding domains of rTLR2 to rLipL32 are the domains that bind other ligands as described previously^35, 36^. This plier-like structure of rLipL32 from the pathogenic *Leptospira* serves as the novel ligand described to interact with rTLR2. Improved understanding of the ligands and binding mechanisms of innate immune components provides further information about how host cells interact with pathogens and suggests potential strategies for drug development.

## Materials and Methods

### Reagents

Fetal calf serum (FCS), Dulbecco’s modified Eagle’s medium (DMEM), and F-12 nutrient mixture (HAM) were obtained from Invitrogen Corp. (Carlsbad, CA). The anti-TLR2 IgG clone T2.5 neutralizing antibody (MAb-mTLR2) and isotype mouse IgG1 were provided by Invivogen (San Diego, CA). The anti-His tag antibody (Ab18184) was purchased from Abcam (Cambridge, MA). All other chemicals were supplied by Sigma (St. Louis, MO).

### DNA construction and fragments deletion

The *lipl32* gene was cloned from pathogenic *L. santarosai* serovar Shermani str. LT821 (ATCC number 43286; pathogenic species) genomic DNA with *pfu*-Turbo DNA polymerase (Stratagene, La Jolla, CA) according to prior investigation^10, 26^. Wild-type and truncated *lipl32* variants were amplified by PCR and inserted into the expression vector pRSET-c (Invitrogen, Groningen, The Netherland). The point mutation variants were performed by Q5® Site-Directed Mutagenesis kit (New England Biolabs, Ipswich, MA). The sense and antisense primers used in this research were listed in Table 1. These cloning products from DNA ligation were transformed into JM109 Competent *E. coli* and the DNA sequences were verified by DNA sequencing.

The human *tlr2* gene was amplified by PCR using the primers listed in Table 1 and the template pUNO-hTLR2 purchased from Invivogen (San Diego, CA). The PCR product was verified by agarose gel electrophoresis and the PCR product was cloned into the pLenti6.3/V5-TOPO lentiviral vector (Invitrogen). Following TOPO ligation, One Shot Stbl3 Chemically Competent *E. coli* were transformed with pLenti6.3/V5-TOPO-TLR2. The real cloned DNA with the right size and right orientation was verified by restriction digests and confirmed by DNA sequencing. The correct clone of p-TLR2-Lenti plasmids was used for stable gene expression in culture HEK293 cells.

### Cell culture

Human embryonic kidney cells (HEK293FT; ATCC® CRL-1573) were maintained in Thermo Scientific HyClone Dulbecco’s Modified Eagle’s Medium (DMEM) supplemented with 10% Thermo Scientific HyClone Defined Fetal Bovine Serum. Cells were grown in an incubator at 37 °C and the humidified atmosphere of 5% CO_2_. All stimulation experiments are performed under serum free conditions to avoid the influence of serum on cell function and investigated events.

### Lentiviral transduction and establishment of stable cell line

The vector p-TLR2-Lenti was transfected into HEK293FT cells with ViraPower packaging mix (pLP1, pLP2, and pLP/vesicular stomatitis virus; ViraPower; Invitrogen) to generate the lentivirus according to manufacturer’s protocol. HEK293FT cells were transfected with the lentivirus and stable cell lines were generated by selecting with blasticidin. Cells were collected and named HEK293-TLR2. Cells were grown in DMEM with 10% FBS and maintained in a 37 °C humidified atmosphere containing 5% CO_2_.

### Protein expression and purification

The DNA plasmid of *lipl32* and its variants were transformed into the expression host cell *E. coli* ClearColiTM^TM^BL21 (DE3) pLys (Lucigen, Middleton, WI). The transformants of His_6_-LipL32 and its variants were grown in Luria broth medium with 50 μg/ml ampicillin at 37 °C to an OD_600_ of ~0.6. Isopropyl 1-thio-β-galactopyranoside (500 μM) was subsequently added to induce the expression of LipL32 protein for additional 24 h at 16 °C. *E. coli* cells were harvested by centrifugation at 4,000 x g for 15 min and then sonicated in phosphate buffered saline (PBS buffer). The cell debris was discarded after centrifugation at 14,000 *xg* for 30 min and the supernatant absorbed to Ni^2+^-nitrilotriacetic acid (Ni^2+^-NTA) agarose resin (GE Healthcare Life Sciences, Chalfont) for affinity chromatography purification^25, 26, 48^. rLipL32 protein and its variants were eluted in PBS buffer containing 250 mM imidazole and the elution fractions were dialysis against the PBS buffer to remove the imidazole and applied to mono Q column ion exchanged column (GE Healthcare Life Sciences, Chalfont) for further removal the contaminated protein and endotoxins. Furthermore, to detach His_6_-tag, the rLipL32 protein and its variants were incubated with 0.2 mg/ml enterokinase (EC 3.4.21.9; Invitrogen) at 37 °C for 16 h^25, 49^. The tag peptide was further removed by size exclusion chromatography using Superdex 200 pg 16/600 column (GE Healthcare Life Sciences, Chalfont). The purified rLipL32 and its variants (100–150 μg) were injected into the column and the flow rate was set at 1 ml/min at 4 °C. To validate the inflammatory effects of rLipL32, the protein were subjected to polymyxin B (Invitrogen), heat, and proteinase K (Invitrogen) treatments, respectively, as described previously^11, 25^. The *Limulus* Amebocyte Lysate (LAL) test was further used to detect the recombinant proteins and measured the endotoxin contamination according to the manufacturer’s guidelines (Lonza, Switzerland).

HEK293-TLR2 was grown in the medium of DMEM with 10% FBS and maintained in a 37 °C humidified atmosphere containing 5% CO_2_ for human rTLR2 protein expression. The cell was suspended in PBS buffer containing 0.5% CHAPS for rTLR2 protein solubilization. The cell debris was removed after centrifugation at 14,000 × g for 30 min and the supernatant absorbed to anti-V5 antibody activated NHS resin (GE Healthcare Life Sciences, Chalfont) for affinity chromatography purification. rTLR2 protein was eluted in 25 mM Glycine-HCl buffer (pH 2.5) and following by neutralization in the PBS buffer containing 0.5% CHAPS.

### Circular dichroism

The secondary structures of rLipL32WT and deletion variants were analyzed at 25 °C on an CD Aviv 202 spectrometer (Aviv Biomedical, Lakewood, NJ) using a 1-mm path length cuvette. The resulting spectra were corrected to remove buffer signal and smoothened using SigmaPlot 10.0. The obtained CD spectra were reported as averages of no less than three scans for each sample.

### Stains-all binding assay

Stains-all is a carbocyanine probe and could be used to detect the Ca^2+^ binding protein. rLipL32WT and its variants (15 μM) were mixed with stains-all dye solution (500 μM) in 2mM MOPS (pH 7.2) containing 30% (v/v) ethylene glycol and incubated for 10 min. The CD spectra was used to scan from 700 to 400 nm and the characteristic peak at 660 nm (J band) was recorded. The obtained CD spectra were reported as averages of no less than three scans for each sample.

### Confocal microscopy

HEK293 and HEK293-TLR2 cells were fixed, permeabilized and incubated with appropriate primary and secondary antibodies: mouse monoclonal anti-TLR2 antibody (eBioscience, San Diego, CA), rabbit monoclonal anti-His_6_ (Research Diagnostics Inc., Cleveland, Ohio). The secondary antibodies for confocal laser scanning microscopy were Alexa594 conjugated anti-mouse and Alexa488 conjugated anti-rabbit secondary antibodies (Research Diagnostics, Inc.). Cells were imaged by confocal laser scanning microscopy (TCS-SP8-X, Leica, Wetzlar, Germany).

### RNA extraction and real-time PCR (RT-PCR)

Total RNA was extracted according to the previous protocol with minor modifications^27, 28^. Real-time PCR was performed following manufacturer’s instructions using an ABI Prism 7700 with SYBR green I as a double-stranded DNA-specific dye (PE-Applied Biosystems, Cheshire, UK). Primers of *IL-8*, *TNF-α*, *MCP-1*, and *hMMP7* were listed in Table 1 and were constructed to be compatible with a single RT-PCR thermal profile (95 °C for 10 min, 40 cycles at 95 °C for 30 s, and 60 °C for 1 min). The accumulation of the PCR product was recorded in real time (PE-Applied Biosystems). In addition, the TaqMan gene expression assays were used to measure *GRO-α* (Hs00236937_m1, Applied Biosystems) and *SPP1* (Hs00959010_m1, Applied Biosystems) mRNA expression. The results of the mRNA levels for the different genes are displayed as transcript levels of the analyzed genes relative to GAPDH. The statistical analyses were performed with Student’s t test. Statistically significant were considered as p-values of ≤0.05.

### Molecular docking

The PDB files corresponding to Ca^2+^-bound (PDB:2WFK^20^) and Ca^2+^-free (PDB:3FRL^19^) LipL32 and TLR2 LRR domain (PDB: 2Z7X, chain A^35^) were submitted to automated docking using the ClusPro 2.0 server (available on-line^40, 50–52^). Each protein was analyzed both as receptor and ligand. The 10 highest scoring models were analyzed using PyMOL (available on-line). The 10 possible binding models were divided in to 3 groups according to the binding domains of LipL32 interacted with TLR2.

### Enzyme-linked immunosorbent assay (ELISA)

The interaction between rLipL32 and rTLR2 was investigated by ELISA according to the prior method with minor modifications^20, 25^. The ELISA plates (Nunc-Immuno Plate MaxiSorp surface) were primarily coated with 1 μg rTLR2 protein in 100 μl PBS buffer and incubated for 2 h at 37 °C. The wells were washed with PBST (PBS containing 0.05% (v/v) Tween 20) and then blocked with 1% (w/v) bovine serum albumin (BSA) for 1 h at 37 °C. The plates were incubated overnight at 4 °C. Protein samples (rLipL32WT and variants; from 0 to 2 μM) in PBS buffer were added to the attached rTLR2 protein for 90 min at 37 °C and then washed with PBST. The bound rLipL32 protein was detected by adding 100 μl anti-His_6_ antibody (1:5,000 dilution) (Proteintech, Rosemont, IL) incubated for 1 h and then washed with PBST. Subsequently, 100 μl horseradish peroxidase conjugated donkey anti-rabbit immunoglobulin G (1:5,000 dilution) was added and incubated for 1 h at 37 °C. After washing with PBST, the 3,3′,5,5′-tetramethylbenzidine (TMB) was added for color development. The reaction was allowed to proceed for 15 min and then terminated by adding 50 μl 2 N H_2_SO_4_. The absorbance was measured at 450 nm in an ELISA reader (BioTek, Winooski, VT). These experiments were accomplished in three independent experiments and the mean absorbance ± S.D. of three independent data sets was calculated. The absorbance at each data point was corrected by subtracting a negative control from BSA coated well. The dissociation constant (*K*_*d*_) according to the prior protocol^53^, based on the following equation:

1 $$A={A}_{max}[{\rm{p}}{\rm{r}}{\rm{o}}{\rm{t}}{\rm{e}}{\rm{i}}{\rm{n}}]/(Kd+[{\rm{p}}{\rm{r}}{\rm{o}}{\rm{t}}{\rm{e}}{\rm{i}}{\rm{n}}])$$

where *A* is the absorbance at a set protein concentration, *A*_max_ is the maximum absorbance for the ELISA plate reader (equilibrium), [protein] is the protein concentration, and *K*_*d*_ is the dissociation equilibrium constant for a given absorbance at a set protein concentration (ELISA data point). The binding data were assayed via SigmaPlot 10.0 program by fitting the data to the optimal equation^54^ assuming that ligands bound to one independent site. The statistical analyses were performed with Student’s t test. Statistically significant were considered as p-values of ≤0.05.

### AFM tip and mica functionalization

Silicon nitride AFM cantilever, PNP-TR (NanoWorld, Neuchâtel, Switzerland), was cleaned by sonication in a series of solvents and functionalization in 3-aminopropyl-trimethoxysilane and glutaraldehyde (Grade II, Sigma) according to the method reported previously with minor modifications^25, 26, 55^. For anchoring proteins, the tips were rinsed with PBS to remove the glutaraldehyde remainders and subsequently incubated with proteins, including BSA, Pam_3_CSK_4_, rLipL32 and its variants for 1 h at room temperature. The functionalized AFM tips were then rinsed three times with PBS to remove the unbound proteins^56^. The mica surface was modified for better deposition of proteins according to previous reports^57, 58^. The unbound proteins were removed and the proteins were fixed on the mica for later AFM measurements.

### AFM observation

The functionalized cantilever tips used in this work had a spring constant in the range of 0.02–0.08 N/m as determined from the amplitude of their thermal vibrations. A commercial atomic force microscope (Nanoscope III, Digital Instruments, Santa Barbara, CA) with a E type scanner was employed throughout this study. The force volume software takes a force curve at each of the 999 points during a two dimensional scan over a sample surface. The X-Y scan size was 150 μm, and the Z scan distance was 5 μm at a rate of 1 Hz. The force applied to the protein modified mica surface was kept below 400 pN. The distance-force curves and force parameters were obtained according to the methods described previously^25, 26^. For antibody neutralization analysis, the protein modified mica was treated with anti-TLR2 antibodies (diluted 1:1,000 in PBS) (Invivogen, San Diego, CA) for 1 h followed by five washes with PBS to remove unbound antibodies. For the binding events, force curves from at least 10 areas on each surface were independently selected for analysis. All the measurements described above were performed with modified tips and showed repeatedly similar results. The statistical analyses were performed with Student’s t test. Statistically significant were considered as p-values of ≤0.05.

### Statistical analysis

All experiments were performed at least three independent processes and the variables are expressed as mean ± SEM and compared by using Student’s t-test or one-way ANOVA. P-values < 0.05 are considered statistically significant. All analyses were performed using the Graphpad Prism 5.1 (Graphpad, La Jolla, CA).

## Electronic supplementary material

Supplemental information
