# Supplementary material for: Active Components of Leptospira Outer Membrane Protein LipL32 to Toll-Like Receptor 2
Source: Sci Rep. 2017 Aug 21;7:8363. doi: 10.1038/s41598-017-08743-y (PMC5566480; doi:10.1038/s41598-017-08743-y)

# Supplementary Information

## Active Components of *Leptospira* Outer Membrane Protein LipL32 to Toll-Like Receptor 2

Shen-Hsing Hsu[1+], Cheng-Chieh Hung[1+], Ming-Yang Chang[1], Yi-Ching Ko[1], Huang-Yu Yang[1], Hsiang-Hao Hsu[1], Ya-Chung Tian[1], Li-Fang Chou[1], Rong-Long Pan[2], Fan-Gang Tseng[3], and Chih-Wei Yang[1]*

[1]Department of Nephrology, Kidney Research Center, Chang Gung Memorial Hospital, Chang Gung University College of Medicine, Taoyuan 33333,

[2]Department of Life Science and Institute of Bioinformatics and Structural Biology, College of Life Science, National Tsing Hua University, Hsin Chu 30013,

[3]Department of Engineering and System Science, College of Nuclear Science, National Tsing Hua University, Hsin Chu 30013, Taiwan, ROC

[+]These authors contributed equally to this work

*Corresponding author:Prof. Chih-Wei Yang, Department of Nephrology, Kidney Research Center, Chang Gung Memorial Hospital, Chang Gung University College of Medicine, 5 Fu-Shing St. Taoyuan 333, Taiwan, ROC. Tel/Fax: +886-3-3281200-2506/+886-3-3282173; E-mail: cwyang@ms1.hinet.net

**The supplementary file contains Figure S1- S5 and the legends**

**Figure S1. The predicted LipL32-TLR2 complex.** The protein complex was predicted from Cluspro website and the top ten scored complex were divided into three groups according to the binding orientation. In model I, LipL32 is predicted to bind TLR2 through the center domain of jellyroll structure. In model II, LipL32 is predicted to interact with TLR2 through N-terminus domain. In model III, LipL32 is predicted to be recognized by TLR2 through N and C termini. Green, LipL32; cyan, TLR2.

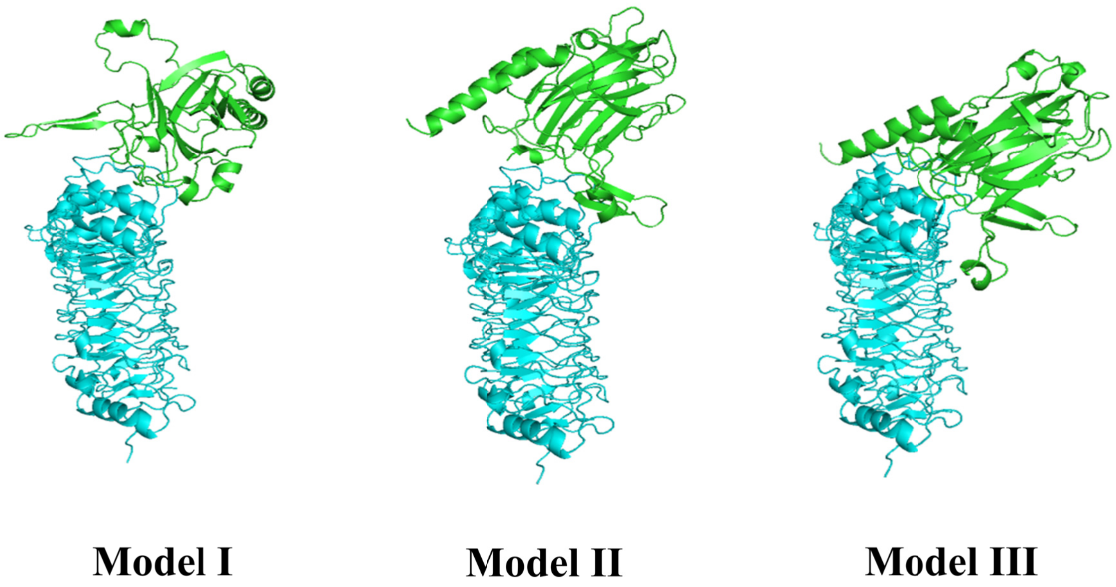

**Figure S2. Purification of human TLR2 protein and LipL32 variants.** (A) Human TLR2 protein was expressed in HEK293-TLR2 cells and purified as described in Materials and Methods. (B) LipL32 protein and its variants were purified as described in Materials and Methods. (C) LipL32 mutation variants were purified as described in Materials and Methods. Upper panel, Western blot; bottom panel, SDS-PAGE.

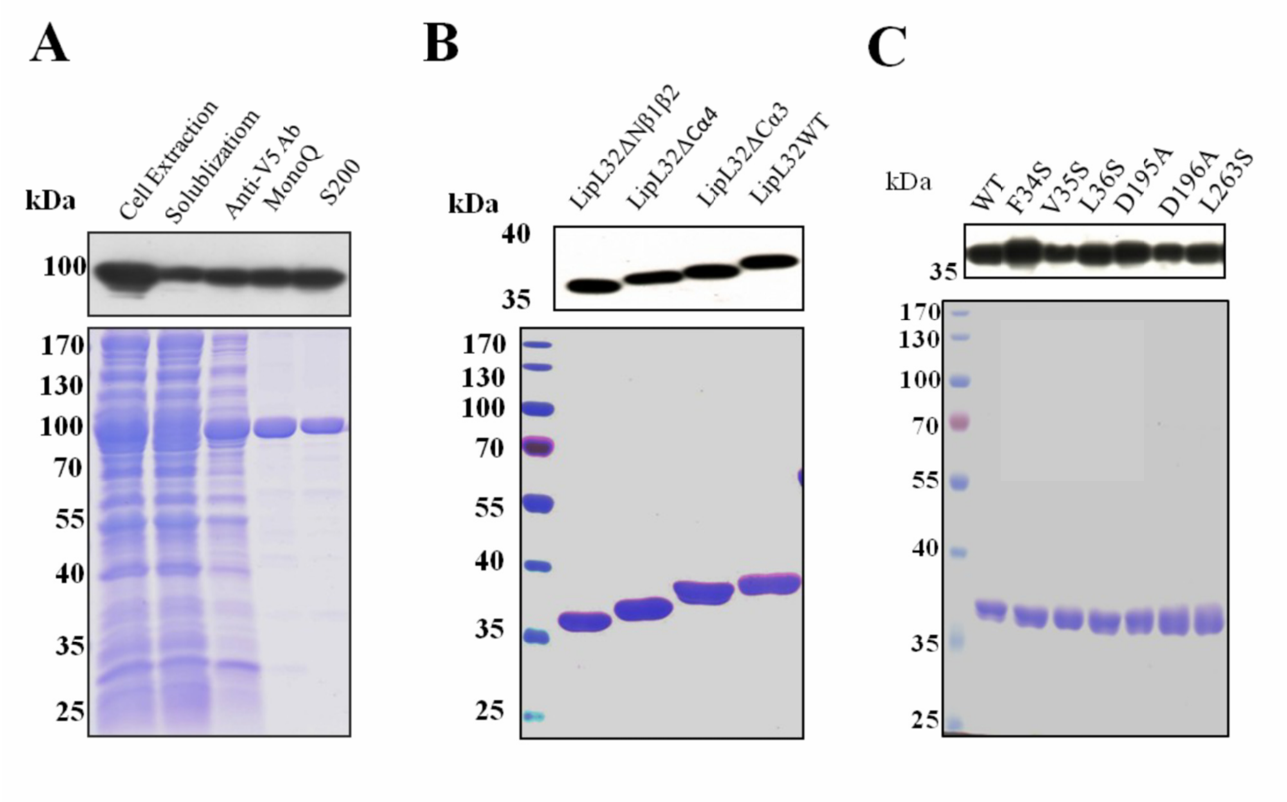

**Figure S3. Functional analysis of purified TLR2 protein.** The function of purified TLR2 protein was tested by the affinity to its nature ligands including lipoteichoic acid (LTA), $Pam_3CSK_4$, and peptidoglycan (PGN), respectively .*, $p<0.05$;**, $p<0.01$;***, $p<0.001$.

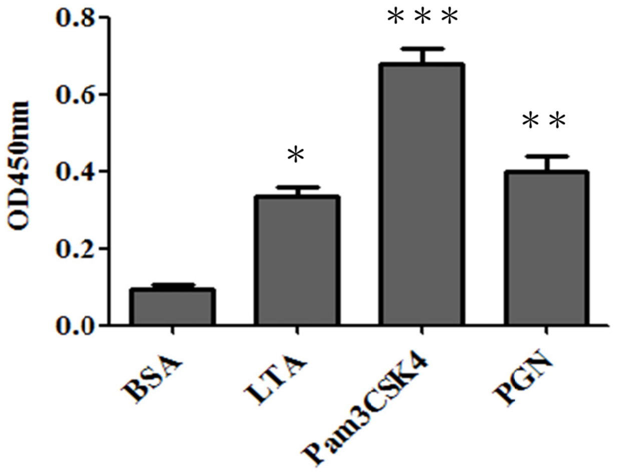

**Figure S4. *Limulus* amebocyte lysate (LAL) assay.** The LAL was used to measure the endotoxin contamination of the recombinant protein from different purification steps, including Ni2+ column, MonoQ column, and polymyxin B treatment, respectively. *, $p<0.05$; ***, $p<0.001$.

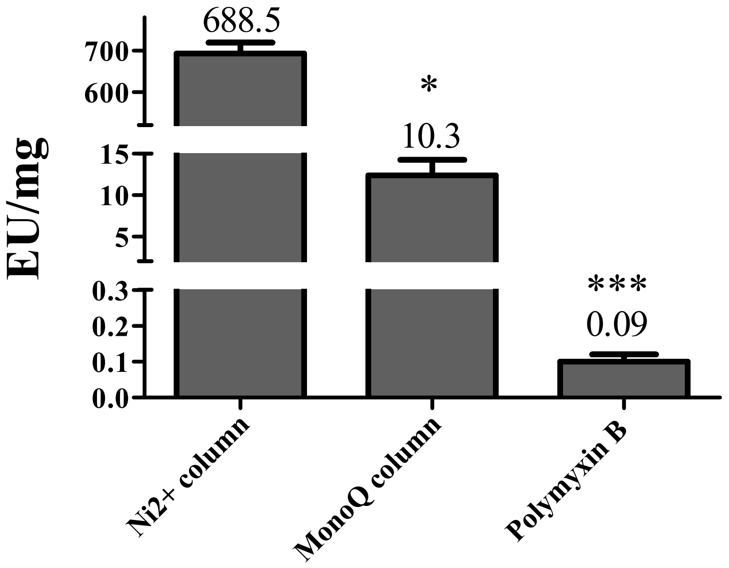

**Figure S5. The stimulation of inflammatory responses by LipL32.** (A) The ability to induce the inflammatory responses of LipL32 in the presence and absence of His6-tag at N terminus. (B) The inflammatory responses induced by LipL32 when the protein treated with heat and proteinase K as compared to WT. *, $p<0.05$.

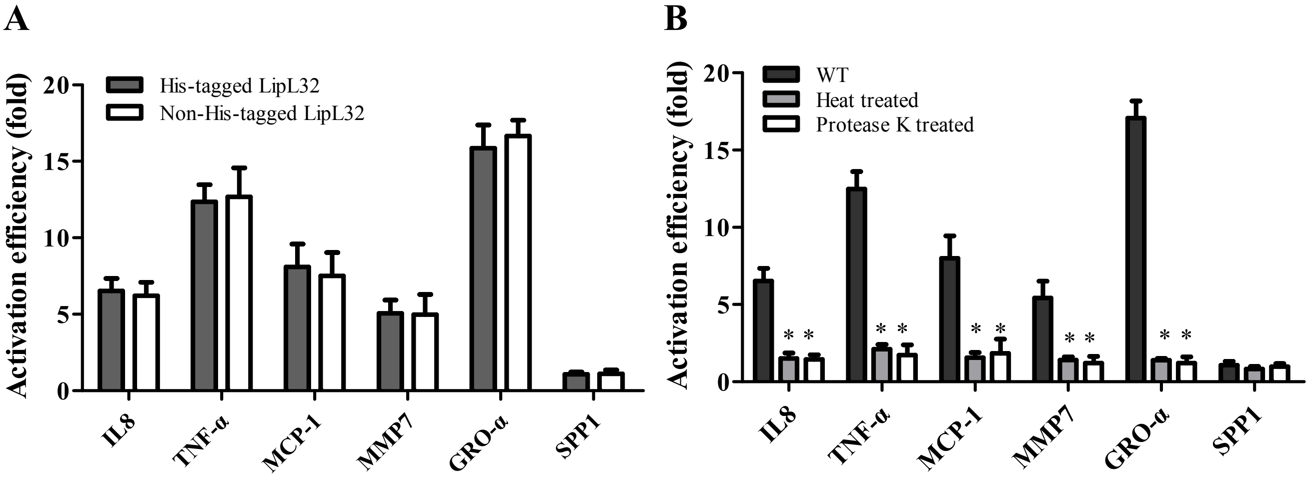

Supplement: Supplementary file 1 — Supplemental information [file 41598_2017_8743_MOESM1_ESM.pdf]
